# Knockout of Sirtuin 3 in endothelial cells impairs endothelial‐dependent relaxation and myogenic response in mice

**DOI:** 10.14814/phy2.70060

**Published:** 2024-10-19

**Authors:** Jian‐Xiong Chen, Jin Zhang, Yingjie Chen, Heng Zeng

**Affiliations:** ^1^ Department of Pharmacology and Toxicology, School of Medicine University of Mississippi Medical Center Jackson Mississippi USA; ^2^ Department of Physiology and Biophysics, School of Medicine University of Mississippi Medical Center Jackson Mississippi USA

**Keywords:** coronary arterioles (CA), EC‐dependent relaxation (EDR), EC‐independent relaxation (EIR), endothelial cell (EC), middle cerebral artery (MCA), myogenic response, sirtuin 3 (SIRT3)

## Abstract

Sirtuin 3 has been shown to regulate endothelial function and coronary flow reserve in mice. Knockout of SIRT3 reduced endothelial nitric oxide synthase expression in the mouse hearts. In this study, we investigate whether endothelial SIRT3 regulates vascular function and myogenic responses in distal intramural branches of the left anterior descending coronary artery (CA) and middle cerebral artery (MCA) of mice. Both male and female endothelial SIRT3 knockout (SIRT3^EC^KO) mice and control SIRT3^LoxP^ mice were used and CA and MCA were dissected and mounted in a myograph system. The myogenic response was evaluated by measuring changes in inner diameter in response to 20 mmHg stepwise increases in intraluminal pressure in PSS (active diameter) and Ca2^+^‐free PSS (passive diameter). Acetylcholine (Ach)‐induced endothelial‐dependent relaxation (EDR) and sodium nitroprusside (SNP)‐induced endothelial‐independent relaxation (EIR) were examined. Our results showed that the myogenic responses were significantly impaired in both the CA and MCA of SIRT3^EC^KO mice. Furthermore, female mice had worsened myogenic response in MCA. In CA, EDR was abolished in both male and female SIRT3^EC^KO mice. Intriguingly, EIR was only reduced in the female mice. In MCA, EDR was reduced in male SIRT3^EC^KO mice, whereas EIR was decreased in both male and female mice. Female SIRT3^EC^KO mice had profound dysfunction in CA, whereas male mice exhibited more dysfunction in MCA. These data revealed a sex and organ‐specific role of endothelial SIRT3 in vascular function and myogenic responses. Our study suggests that endothelial SIRT3 is necessary for maintaining vascular function and blood flow autoregulation.

## 1 INTRODUCTION

Sirtuins belong to a conserved family of histone/protein deacetylases that require the cofactor nicotinamide adenine dinucleotide (NAD+) to deacetylate lysine residues in proteins (Giralt & Villarroya, 2012; McDonnell et al., 2015; Park et al., 2011). Sirtuins mediate histone protein posttranslational modification by coupling lysine deacetylation to NAD^+^ hydrolysis. These enzymes are indispensable in regulating various cellular processes such as energy metabolism, reactive oxygen species (ROS), and cell survival (Tanno et al., 2012). Among the sirtuins, SIRT3 is mainly localized in the mitochondria of cardiac cells, it regulates mitochondrial function and cellular metabolism (He et al., 2019; Zeng & Chen, 2019). Sirtuin 3 (Sirt3) has emerged as a protein of particular interest to human aging and heart diseases (Giralt & Villarroya, 2012; McDonnell et al., 2015; Park et al., 2011). The levels of SIRT3 have been associated with longevity in humans, and protection against aging, cardiac hypertrophy, and oxidative stress (Pillai et al., 2010; Sack, 2012; Zeng et al., 2020a; Zhou et al., 2022). Our previous studies have shown that decreased SIRT3 levels cause coronary microvascular dysfunction, impaired coronary flow reserve (CFR), diastolic function, and cardiac recovery post‐myocardial ischemia (He et al., 2016, 2017). We also found that the knockout of SIRT3 caused an imbalanced arginase II and endothelial nitric oxide synthase (eNOS) with impaired CFR in mouse hearts. The CFR was improved by rebalanced arginase II/eNOS, which is the potential mechanism of SIRT3 KO‐induced coronary microvascular dysfunction (Su et al., 2020). These studies suggest that endothelial SIRT3 may play an important role in vascular function. However, the direct role of endothelial SIRT3 on endothelial‐dependent relaxation (EDR) or endothelial‐independent relaxation (EIR) has not been studied yet. In addition, our previous study demonstrates a sex‐different role of endothelial SIRT3 in regulating blood pressure and coronary function in female mice (Zeng et al., 2020a). To extend this finding, we will further investigate whether there is any sex‐specific role of endothelial SIRT3 on vascular function and myogenic response.

The myogenic response is a fundamental mechanism that regulates blood flow in response to changes in intraluminal pressure. This response is critical for maintaining adequate perfusion to organs and tissues, such as skeletal muscle, intestine, brain, kidney, and heart (Izzard & Heagerty, 2014; Johnson, 1989; Loutzenhiser et al., 2002; Ochi et al., 2023). Furthermore, various vascular beds have different myogenic responses in small arteries. The myogenic responses are greater in cerebral and renal arteries than in other organ arteries such as mesenteric and hindlimb arteries (Just & Arendshorst, 2005; Lagaud et al., 1999). Accumulating evidence revealed that the impaired myogenic response has contributed to the development of vascular damage or an end organ failure related to hypertension, heart failure, and cerebral dysfunction (Cipolla & Curry, 2002; Kold‐Petersen et al., 2012; Ledoux et al., 2003; Schofield et al., 2002). For instance, the myogenic response to coronary arteriole is particularly critical because it regulates blood flow and is essential for normal myocardial blood supply. The impairment of coronary myogenic response may cause a mismatch of myocardial blood supply and demand, which contributes to ischemic heart diseases (Kroetsch & Bolz, 2013). In hypertension, the myogenic response is impaired due to the abnormal response of vascular smooth muscle cells to blood pressure changes (Izzard & Heagerty, 1995). This leads to reduced vascular compliance and increased vascular resistance, which contributes to the development and progression of hypertension. In heart failure, the myogenic response is also impaired, which can exacerbate cardiac dysfunction (Hoefer et al., 2010). Although impairment of myogenic response has been extensively studied, the underlying molecular mechanisms are largely unknown. Endothelial cells in the blood vessel's inner layer are the first organ to react virtually immediately in response to changes in intraluminal pressure. Although myogenic responses originate in vascular smooth muscle, its contractile function may be modulated by endothelium via releasing vascular contractive factors. A recent study highlights the critical role of endothelial mitochondrial oxidative phosphorylation in the control of vascular tone. Furthermore, the requirement for mitochondrial ATP production on vascular tone is independent of sex and vascular beds (Harraz OF, 2023; Wilson et al., 2023). Although endothelial SIRT3 regulates mitochondrial oxidation and ATP production, the sex and organ‐specific role of endothelial SIRT3 on vascular function and the myogenic response has not been investigated. In thís study, we test whether the specific knockout of endothelial mitochondrial SIRT3 will impair endothelial‐dependent relaxation (EDR), and endothelial‐independent relaxation (EIR) and alter the myogenic response of coronary arterioles and cerebral arterioles in a sex and organ‐specific manner.

## 2 METHODS

### 2.1 Mice

SIRT3^LoxP^ (SIRT3 LoxP) mice were obtained from Dr. Eric Verdin at Gladstone Institute of Virology and Immunology, University of California, and used as corresponding controls. SIRT3^LoxP^ mice were crossed with VE‐Cadherin‐Cre (Cdh5‐Cre) transgenic mice (B6.FVB‐Tg[Cdh5‐cre]7MLia/J [Strain #006137, Jackson Laboratory]) to generate an endothelial‐specific SIRT3 knockout (SIRT3^EC^KO) mouse strain as described previously (He et al., 2017). Endothelial SIRT3 absence was confirmed by our previous study (He et al., 2017). Both male and female SIRT3^EC^KO mice and their respective control SIRT3^LoxP^ mice at the age of 12–15 months were used for experiments. All animals were fed with laboratory standard chow (Teklad rodent diet, non‐autoclavable form, catalogue number: 8604, Madison, Wisconsin) and water and housed in individually ventilated cages in the Laboratory Animal Facilities at the University of Mississippi Medical Center. All protocols were approved by the Institutional Animal Care and Use Committee (IACUC) of the University of Mississippi Medical Center (Protocol ID: 1189 and 1596) and were consistent with the National Institutes of Health Guide for the Care and Use of Laboratory Animals (NIH Pub. No. 85–23, Revised 1996).

### 2.2 Materials

All chemicals were purchased from Sigma‐Aldrich. The compositions of physiological salt solution (PSS) contained (in mmol/L) 119 NaCl, 4.7 KCl, 1.17 MgSO_4_, 1.6 CaCl_2_, 18 NaHCO_3_, 5 HEPES, 1.18 NaH_2_PO_4_, and 10 glucose. pH was adjusted to 7.4. Calcium‐free physiologic salt solution (PSS_0Ca_) was PSS without CaCl_2_ and with the addition of EDTA (0.03 mM). Acetylcholine (Ach, catalogue number: 159171000, Thermo scientific) was used for testing endothelial‐dependent relaxation (EDR) (Si et al., 2020). The dissolved concentrations of acetylcholine (Ach) were 10^−9^–10^−4^ M. Sodium nitroprusside (SNP, NO donor, catalogue number: 152061, MP Biomedicals, Solon, Ohio) was used for testing endothelial‐independent relaxation (EIR) (Juguilon et al., 2022). The dissolved concentration of SNP was 10^−9^–10^−5^ M. U‐46619 (a selective agonist of prostaglandin H_2_/thromboxane A_2_ receptor, catalogue number:16450, Cayman Chemical Company) was used to induce vasoconstriction before testing EDR and EIR (Sabe et al., 2024). The dissolved concentration of U46619 was 10^−6^ M. *N*_ω_‐Nitro‐L‐arginine methyl ester hydrochloride (L‐NAME, catalogue number: 80210, Cayman Chemical Company), a NO synthase inhibitor, was used to block endogenous NO production before testing EIR (Si et al., 2020). The dissolved concentration of L‐NAME was 4 mM. All chemicals were prepared as stock solutions in PSS.

### 2.3 Dissection of coronary arterioles and middle cerebral arteries

Mice were killed with 4% isoflurane and the hearts or brains were collected and immediately placed in a dish filled with ice‐cold (4°C) PSS. The distal intramural branches of the left anterior descending coronary artery (CA) of an inner diameter of 25–80 μm or the middle cerebral artery (MCA) of an inner diameter of 50–120 μm were dissected under a microscope (Amscope, MU1803, Irvine, CA) (Figure S1a).

### 2.4 Cannulated artery preparation and pressure myography

The CA or MCA was placed on glass microcannulas in a chamber filled with 37°C PSS at pH 7.4 and aerated with the gas mixture (95% O_2_–5% CO_2_) in the Pressure Myograph System 114P (Danish Myo Technology, Denmark, Figure S1b). The pressure servo‐controlled myograph system consists of a stainless chamber with proximal and distal cannulas. The artery was mounted on a proximal cannula and tied properly by a nylon thread. Another end of the artery was tied onto the distal cannula firmly. The arterial lumen was filled with the same PSS that filled the chamber. Branches of the artery were tied off to prevent leakage. The distance between the cannular tips was adjusted with a micrometer connected to the proximal cannula to remove the looseness of the artery. The diameter of the artery was monitored continuously with a video system containing a digital camera attached to the inverted microscope Zeiss Axio Vert. A1 and a computer monitor. Vessel diameters were measured with a video scaler (AmScope MyoVIEW5 Acquisition software). The video scaler was calibrated with a micrometer scale. Each artery was allowed to equilibrate for 30 min at an intraluminal pressure of 70 mmHg to establish basal tone. After the establishment of basal tone, the viabilities of the artery were tested by contraction with KCl (60 mM).

To examine the pressure‐flow‐induced myogenic response of the artery, the changes in the inner and outer diameter (ID and OD) were measured in response to intraluminal pressure increases from 10 to 150 mmHg in a 20 mmHg stepwise manner to determine the passive diameters. To test the mechanical properties of the artery, such as wall thickness, cross−sectional area, wall‐to‐lumen ratio, and distensibility, the intraluminal pressure was reset to 5 mmHg, and the chamber was replaced with calcium‐free PSS (PSS_0Ca_) at the end of the experiments. The inner and outer diameters of the artery under calcium‐free conditions (ID_0Ca_ and OD_0Ca_) were measured at 5 mmHg or intraluminal pressure increasing from 10 to 150 mmHg in a 20 mmHg stepwise manner to determine passive diameters.

### 2.5 Vascular reactions for drug treatments

These studies were performed in the arteries of control SIRT3^LoxP^ mice and SIRT3^EC^KO mice with intraluminal pressure at 70 mmHg. This series of studies examined Ach‐induced endothelial‐dependent relaxation (EDR) in CA or MCA. Briefly, U46619 (10^−6^ M) was used to induce maximum precontraction. Then, vessels were incubated with different concentrations of Ach (10^−9^–10^−4^ M) to induce EDR. The alterations in the inner diameter of the CA or MCA were measured. The percentage of vessel relaxation to maximum precontraction was calculated. The CA or MCA were then washed with PSS and allowed to return to basal tone before testing SNP‐induced endothelial‐independent relaxation (EIR). The CA or MCA was incubated with L‐NAME (4 mM) for 1 h to blockade endogenous NO production. Then the U46619 (10^−6^ M) was used to induce precontraction again. After the maximum contraction, SNP (10^−9^–10^−5^ M) was added to evaluate endothelial‐independent relaxation (EIR). The percentage of vessel relaxation to maximum precontraction was calculated.

### 2.6 Calculation of mechanical properties of arteries

The following mechanical properties of arteries were calculated using equations described previously (Briones et al., 2003; Cheng et al., 2014; Izzard et al., 2003):

Wall thickness (𝜇m) = (OD_0Ca_ − ID_0Ca_)/2.

Cross‐sectional area (CSA, 𝜇m^2^) = (𝜋/4) × (OD^2^_0Ca_ − ID^2^_0Ca_).

Wall to lumen ratio = Wall thickness/ID_0Ca_.

Myogenic tone (%) = [(ID_0Ca_ − ID)/ID_0Ca_] × 100.

Distensibility (%) = (ID_0Ca_ − ID_0Ca 5mmHg_)/ID_0Ca 5mmHg_ × 100.

where ID_0Ca5mmHg_ was the inner diameter obtained at the perfusion pressure of 5 mmHg in PSS_0Ca_.

Circumferential wall strain (*𝜀*) = (ID_0Ca_ − ID_0Ca 5mmHg_)/ID_0Ca 5mmHg_.

Circumferential wall stress (*𝜎*) = (*P* × ID_0Ca_)/2 × wall thickness.

where *P* was intraluminal pressure (1 mmHg = 133.4 Nm^−2^) under calcium‐free conditions.

Arterial stiffness was defined as the ability of the artery to resist elastic deformation when subjected to pressure. It was determined by elastic modulus (*E* = Circumferential wall stress [*𝜎*]/Circumferential wall strain [*𝜀*]). The relationship of *𝜎* and *𝜀* was nonlinear and appropriate to the exponential curve. Thus, an exponential model with least‐squares analysis was used: *𝜎*=*𝜎*_orig_e^𝛽𝜀^.

where σ_orig_ was defined as *σ* at the original diameter of perfusion pressure 5 mmHg. The slope of the curve (*β* value) was used to determine the tangential or incremental elastic modulus (*E*_inc_), which was directly proportional to *E*_inc_. An increased *β* value indicated an increase in stiffness.

### 2.7 Statistical analysis

Data are presented as mean values ± standard deviation (SD). Statistical analysis was performed using Student's unpaired two‐tailed *t*‐test for comparisons between two groups. Analysis was conducted using GraphPad Prism 10 (GraphPad Software, Inc.). A *p* < 0.05 was considered to be significant.

## 3 RESULTS

### 3.1 The effects of endothelial SIRT3 deficiency on endothelial‐dependent relaxation (EDR) and endothelial‐independent relaxation (EIR) in coronary arteries

**FIGURE 1:**
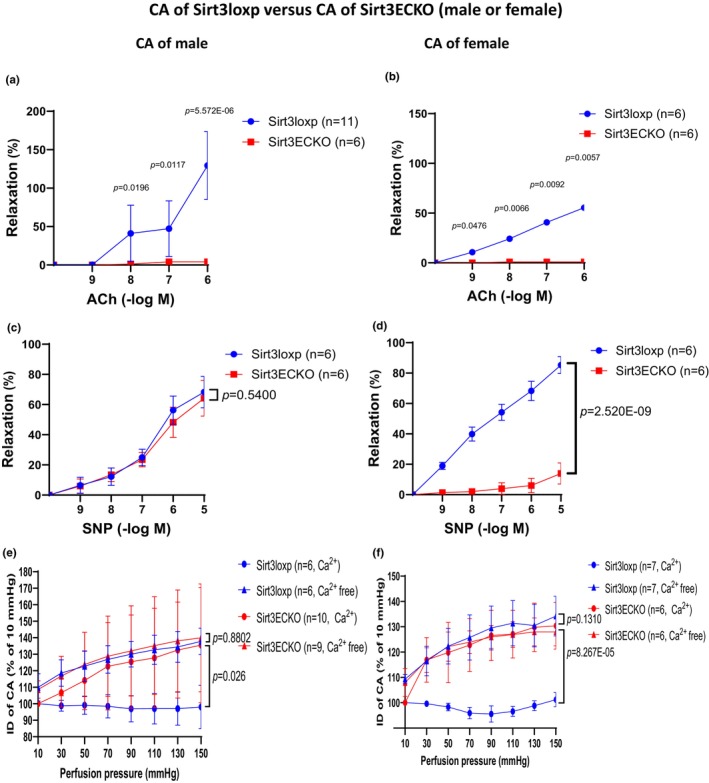
(a) Ach‐induced vessel relaxation after U46619 (1 μM) precontraction in CA of male mice. Ach‐induced EC‐dependent relaxation was significantly reduced in the coronary artery (CA) of male Sirt3^EC^KO mice. (*N* = 6–11, Mean ± SD). (b) Ach‐induced vessel relaxation after U46619 (1 μM) precontraction in CA of female mice. Ach‐induced EC‐dependent relaxation was abolished in the CA of female Sirt3^EC^KO mice versus female control mice. (*N* = 6, Mean ± SD). (c) SNP‐induced vessel relaxation after U46619 (1 μM) precontraction in CA of male mice. SNP‐induced endothelium‐independent relaxation was unaltered in the CA of male Sirt3^EC^KO versus control mice. (N = 6, Mean ± SD). (d) SNP‐induced vessel relaxation after U46619 (1 μM) precontraction in CA of female mice. SNP‐induced endothelium‐independent relaxation was significantly reduced in the CA of female Sirt3^EC^KO mice. (N = 6, Mean ± SD). (e) The perfusion pressure‐induced myogenic response and compliance in CA of male mice. Data representing the comparison of IDs of control mice versus Sirt3^EC^KO mice in PSS, and the comparison of IDs of control mice versus Sirt3^EC^KO mice in Ca^2+^‐free PSS. The myogenic response in CA of male Sirt3^EC^KO mice was impaired and the compliance of male Sirt3^EC^KO mice was unchanged. (*N* = 6–10, Mean ± SD). (f) The perfusion pressure‐induced myogenic response and compliance in CA of female mice. Data representing the comparison of IDs of control mice versus Sirt3^EC^KO mice in PSS, and the comparison of IDs of control mice versus Sirt3^EC^KO mice in Ca^2+^ − free PSS. The myogenic response in CA of female Sirt3^EC^KO mice was significantly impaired compared to control female mice. The compliance of female Sirt3^EC^KO mice was unaltered as compared to control female mice. (*N* = 6–7, Mean ± SD).

We first tested endothelial‐dependent relaxation (EDR) in CA by incubating with Ach (10^−9^–10^−4^ M) after U46619 precontraction in SIRT3^EC^KO mice and control SIRT3^LoxP^ mice. As shown in Figure 1a,b, treatment of vessels with Ach (10^−9^–10^−4^ M) resulted in a dose‐dependent relaxation in the CA of control SIRT3^LoxP^ mice. Ach‐induced EDR was significantly reduced in the CA of male SIRT3^EC^KO mice as compared to male SIRT3^LoxP^ mice. The female SIRT3^EC^KO mice also exhibited a significant reduction of EDR. Furthermore, Ach‐induced EDR was abolished in female SIRT3^EC^KO mice (Figure 1b).

Next, we tested endothelial‐independent relaxation (EIR) in CA by incubating with NO donor SNP (10^−9^–10^−5^ M) after U46619 precontraction. Treatment of vessels with SNP (10^−9^–10^−5^ M) resulted in a dose‐dependent relaxation in the CA of control SIRT3^LoxP^ mice (Figure 1c,d). Interestingly, SNP‐induced EIR had no differences between the male SIRT3^EC^KO mice and control mice (Figure 1c). However, SNP‐induced EIR was significantly reduced in female SIRT3^EC^KO mice (Figure 1d), which indicates that the knockout of SIRT3 in endothelium may disrupt the function of vascular smooth muscle (VSMC), and suggests an EC‐VSMC interaction in female CA.

### 3.2 Knockout of endothelial SIRT3 impaired pressure‐flow‐induced myogenic response in coronary arteries

In the CA of control SIRT3^loxP^ mice, the inner diameter (ID) was reduced in response to 20 mmHg stepwise increases in intraluminal pressure in the PSS (active diameter). Whereas the ID was increased in response to 20 mmHg stepwise increases in intraluminal pressure in the Ca2 + −free PSS (passive diameter) (Figure 1e,f). In the CA of SIRT3^EC^KO mice, increasing perfusion pressure led to a vessel dilation of CA either with or without Ca^2+^ in PSS (Figure 1e,f), suggesting male SIRT3^EC^KO mice had an impaired myogenic response without affecting vessel compliance in CA. Similar results were observed in female SIRT3^EC^KO mice (Figure 1f). The wall thicknesses and cross‐sectional area (CSA) were significantly increased in SIRT3^EC^KO male mice compared to control SIRT3^LoxP^ male mice (Figure S2a,c), while there were no difference between female SIRT3^EC^KO and SIRT3^LoxP^ mice (Figure S2b,d). However, the wall‐to‐lumen ratios in CA were not different (Figure S2e,f). In addition, the wall tensions and myogenic tones in CA of SIRT3^EC^KO mice were significantly reduced (Figure S3a–d). The distensibility and stiffnesses in CA were not altered by SIRT3^EC^KO (Figure S4a–f).

### 3.3 Knockout of endothelial SIRT3 reduced endothelial‐dependent relaxation (EDR) and endothelial‐independent relaxation (EIR) in middle cerebral arteries

**FIGURE 2:**
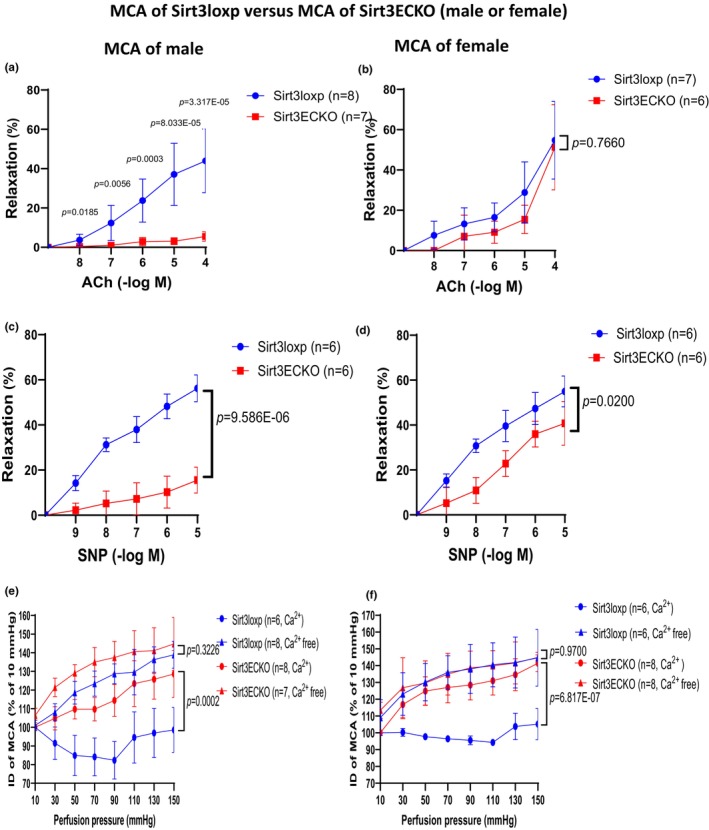
(a) Ach‐induced vessel relaxation after U46619 (1 μM) precontraction in the middle cerebral artery (MCA) of male mice. Ach‐induced EC‐dependent relaxation was significantly reduced in the MCA of male Sirt3^EC^KO mice. (*N* = 7–8, Mean ± SD). (b) Ach‐induced vessel relaxation after U46619 (1 μM) precontraction in MCA of female mice. Ach‐induced EC‐dependent relaxation was unaltered in the MCA of female Sirt3^EC^KO mice. (*N* = 6–7). (c) SNP‐induced vessel relaxation after U46619 (1 μM) precontraction in MCA of male mice. SNP‐induced EC‐independent relaxation was impaired in MCA of male Sirt3^EC^KO mice. (*N* = 6, Mean ± SD). (d) SNP‐induced vessel relaxation after U46619 (1 μM) precontraction in MCA of female mice. SNP‐induced endothelium‐independent relaxation was impaired in the MCA of female Sirt3^EC^KO mice. (*N* = 6, Mean ± SD). (e) The perfusion pressure‐induced myogenic response and compliance in MCA of male mice. Data representing the comparison of inner diameters (IDs) of male control versus male Sirt3^EC^KO mice in physiologic salt solution (PSS) as well as comparison of IDs of male control versus Sirt3^EC^KO mice in calcium‐free physiologic salt solution (Ca^2+^‐free PSS). The myogenic response in MCA of male Sirt3^EC^KO mice was significantly reduced compared to control male mice. The compliance in MCA of male Sirt3^EC^KO was unaltered compared to control male mice. (*N* = 6–8, Mean ± SD). (f) The perfusion pressure‐induced myogenic response and compliance in MCA of female mice. Data representing the comparison of IDs of female control versus female Sirt3^EC^KO mice in PSS and comparison of IDs of female control versus female Sirt3^EC^KO mice in Ca^2+^‐free PSS. The myogenic response in MCA of female Sirt3^EC^KO mice was significantly impaired compared to control female mice. The compliance in MCA of female Sirt3^EC^KO mice was unaltered. (*N* = 6–8, Mean ± SD).

Treatment with Ach (10^−9^–10^−4^ M) led to a dose‐dependent relaxation in the MCA of control SIRT3^LoxP^ mice. Ach‐induced EDR was significantly reduced in the MCA of male, but not female SIRT3^EC^KO mice (Figure 2a,b). Similarly, incubation of vessels with SNP (10^−9^–10^−5^ M) resulted in a dose‐dependent relaxation in the MCA of control SIRT3^LoxP^ mice. Interestingly, SNP‐induced EIR was significantly reduced in both male and female SIRT3^EC^KO mice as shown in Figure 2c,d.

### 3.4 Knockout of endothelial SIRT3 impaired pressure‐flow‐induced myogenic response in middle cerebral arteries

In MCA, the myogenic responses were significantly reduced without alteration of vessel compliance in both males and females (Figure 2e,f). The wall thicknesses of MCA were slightly higher in males but exhibited dramatically higher in female SIRT3^EC^KO mice than in control mice (Figure S5a,b). The MCA cross‐sectional area and wall‐to‐lumen ratio were significantly higher in SIRT3^EC^KO female mice than in female control SIRT3^LoxP^, but there was no difference in males (Figure S5c,f). The wall tensions and myogenic tones in MCA of SIRT3^EC^KO mice were also significantly reduced (Figure S6a–d). The distensibility and stiffnesses in MCA were not altered by SIRT3^EC^KO (Figure S7a–e).

### 3.5 Sex and organ‐specific effect of endothelial SIRT3 on endothelial‐dependent relaxation (EDR) and endothelial‐independent relaxation (EIR)

**FIGURE 3:**
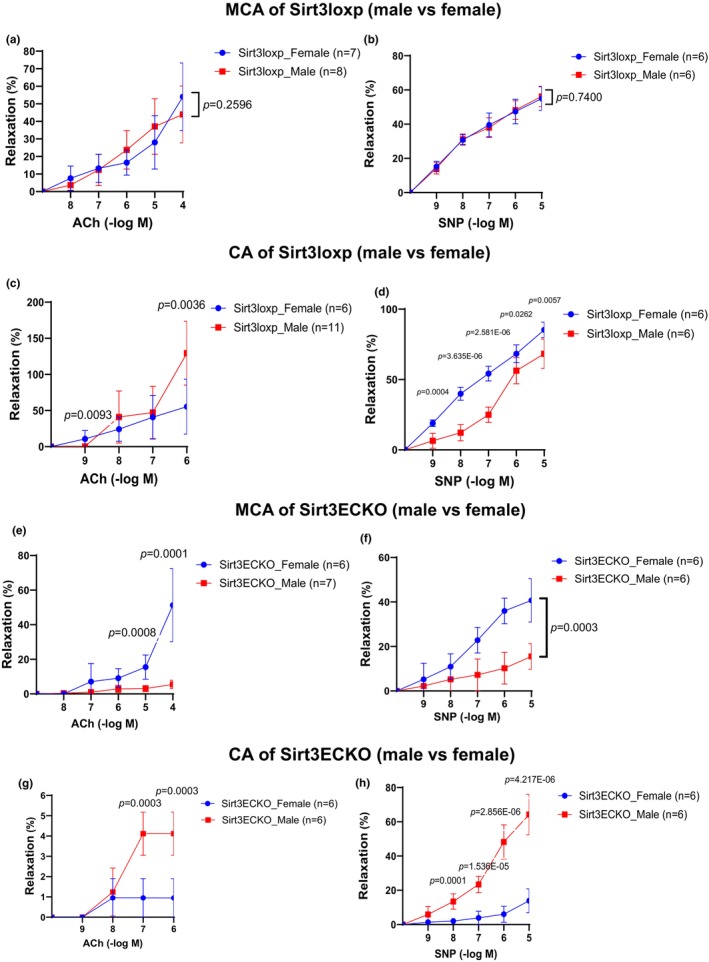
(a) Ach‐induced vessel relaxation after U46619 (1 μM) precontraction in MCA of male and female control SIRT3^LoxP^ mice. Ach‐induced endothelium‐dependent relaxation was not significantly different in the MCA of male versus female control mice. (N = 7–8, Mean ± SD). (b) SNP‐induced vessel relaxation after U46619 (1 μM) precontraction in MCA of male and female control SIRT3^LoxP^ mice. SNP‐induced endothelium‐independent relaxation was unchanged in MCA of female versus male control mice. (*N* = 6, Mean ± SD). (c) Ach‐induced vessel relaxation after U46619 (1 μM) precontraction in CA of male and female control SIRT3^LoxP^ mice. Ach‐induced endothelium‐dependent relaxation in the CA of male control mice was better than the CA of female control mice. (*N* = 6–11, Mean ± SD). (d) SNP‐induced vessel relaxation after U46619 (1 μM) precontraction in CA of male and female control SIRT3^LoxP^ mice. SNP‐induced endothelium‐independent relaxation in CA of female control mice was better than in male control mice. (*N* = 6, Mean ± SD). (e) Ach‐induced vessel relaxation after U46619 (1 μM) precontraction in MCA of male and female Sirt3^EC^KO mice. Ach‐induced endothelium‐dependent relaxation in MCA of male Sirt3^EC^KO mice was significantly reduced, compared to female Sirt3^EC^KO mice. (N = 6–7, Mean ± SD). (f) SNP‐induced vessel relaxation after U46619 (1 μM) precontraction in MCA of male and female Sirt3^EC^KO mice. SNP‐induced endothelium‐independent relaxation in MCA of male Sirt3^EC^KO mice was significantly reduced compared to female Sirt3^EC^KO mice. (N = 6, Mean ± SD). (g) Ach‐induced vessel relaxation after U46619 (1 μM) precontraction in CA of male and female Sirt3^EC^KO mice. Ach‐induced endothelium‐dependent relaxation in CA of female Sirt3^EC^KO mice was significantly reduced compared to male Sirt3^EC^KO mice. (N = 6, Mean ± SD). (h) SNP‐induced vessel relaxation after U46619 (1 μM) precontraction in CA of male and female Sirt3^EC^KO mice. SNP‐induced endothelium‐independent relaxation in CA of female Sirt3^EC^KO mice was reduced compared to male Sirt3^EC^KO mice. (N = 6, Mean ± SD).

To further investigate the sex differences of endothelial SIRT3 on vascular function, we first compared the EDR and EIR in MCA of male and female control SIRT3^LoxP^ mice. There were no significant differences between male and female control mice (Figure 3a,b). Interestingly, Ach‐induced EDR had better relaxation in the CA of males than in female mice (Figure 3c). In contrast, the EIR was significantly less in the CA of male mice compared to female control mice (Figure 3d).

In the comparison of male and female SIRT3^EC^KO mice, the Ach‐induced EDR was less in the MCA of male mice as compared to the female mice (Figure 3e). The SNP‐induced EIR in MCA was also significantly reduced in males as compared to that of females (Figure 3f). In CA, Ach‐induced EDR was reduced in female mice as compared to male mice (Figure 3g). Similarly, the SNP‐induced EIR was significantly reduced in the CA of females compared to males (Figure 3h).

### 3.6 Sex‐specific effect of endothelial SIRT3 on myogenic responses and mechanical properties

**FIGURE 4:**
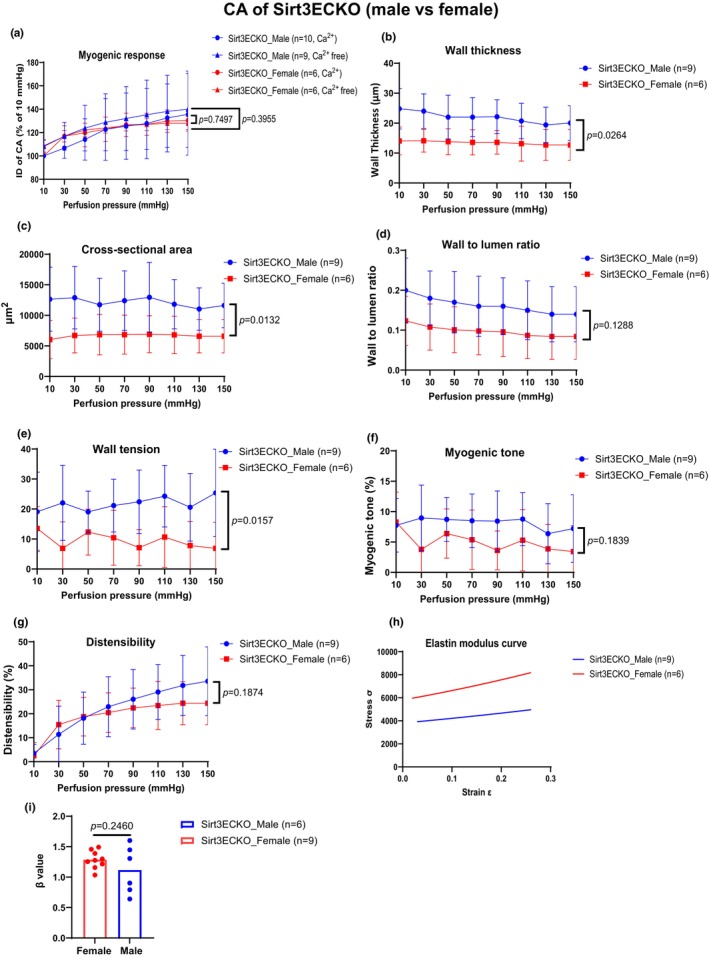
The perfusion pressure‐induced myogenic response and mechanical properties in CA of male and female Sirt3^EC^KO mice. (a) The perfusion pressure‐induced myogenic response had a similar reduction in the CA of male and female SIRT3^EC^KO mice. (N = 6–10, Mean ± SD). (b–e) The CA of male Sirt3^EC^KO mice exhibited significant increases in wall thickness, cross‐sectional area, and wall tension as compared with female Sirt3^EC^KO mice. (N = 6–9, Mean ± SD). (f–g) The myogenic tone and distensibility showed slight increases in male Sirt3^EC^KO mice, but these changes did not reach statistical significance. (N = 6–9, Mean ± SD). (h–i) There is an increase in stiffness (elastin modulus curve and *β* value) in CA of the female Sirt3^EC^KO mice compared to male Sirt3^EC^KO mice, but these changes did not reach statistical significance. (N = 6–9, Mean ± SD).

Both male and female SIRT3^EC^KO mice exhibited a similar impairment of the pressure‐flow‐induced myogenic responses in CA (Figure 4a). The wall thickness, cross‐sectional area, and wall tension in CA were significantly increased in male SIRT3^EC^KO mice as compared to female SIRT3^EC^KO mice (Figure 4b,c,e), suggesting male mice had profound alterations of vessel mechanical properties. The wall‐to‐lumen ratio, myogenic tone, distensibility, and stiffness in CA were not significantly different between male and female SIRT3^EC^KO mice (Figure 4d,f–i). In addition, there were no significant differences in the CA mechanical properties between control SIRT3^LoxP^ male and female mice (Figure S8a,b,d–i), except the cross‐section area (Figure S8c).

**FIGURE 5:**
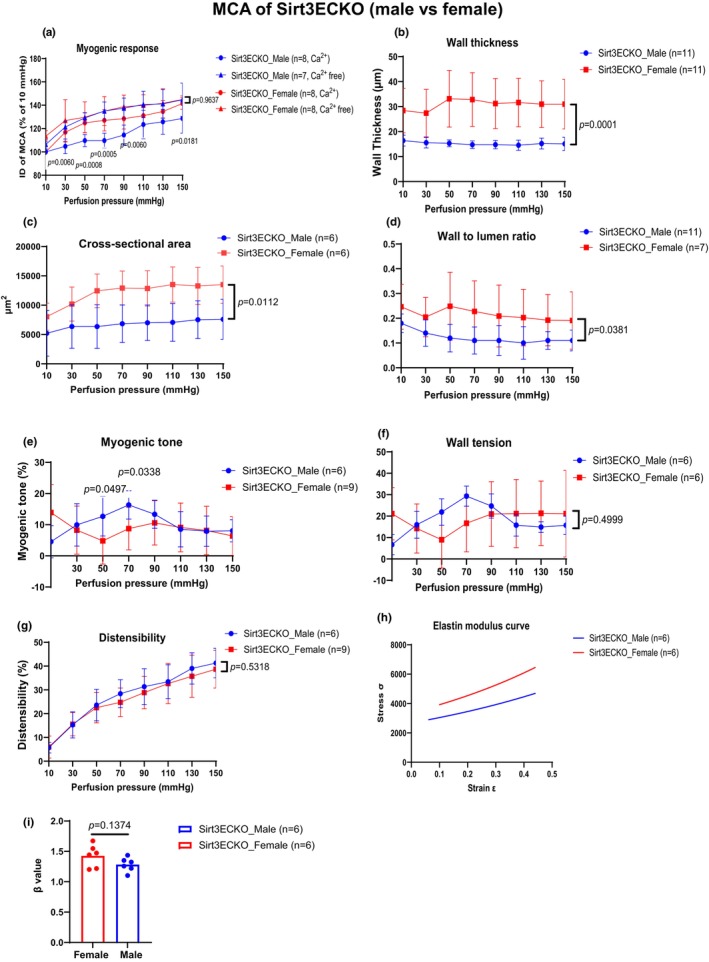
The perfusion pressure‐induced myogenic response and compliance in MCA of male and female Sirt3^EC^KO mice. (a) and (e) Male Sirt3^EC^KO mice revealed an increase in myogenic response and myogenic tone compared to females, especially at 50–70 mmHg perfusion pressure range. (N = 6–9, Mean ± SD). (b–d) Female SIRT3^EC^KO mice revealed an increase in wall thickness, cross‐sectional area, and wall‐to‐lumen ratio compared to male SIRT3^EC^KO mice. (N = 6–11, Mean ± SD). (f–i) MCA revealed similar wall tensions, distensibility, and stiffness in response to perfusion pressure in male and female Sirt3^EC^KO mice. (N = 6–11, Mean ± SD).

Interestingly, the pressure‐flow‐induced myogenic responses were significantly impaired in MCA of female SIRT3^EC^KO mice than that of male SIRT3^EC^KO mice when the perfusion pressure was increased from 30 to 90 mmHg (Figure 5a). The wall thickness, CSA, and wall to lumen ratio in MCA were significantly increased in female SIRT3^EC^KO mice as compared to male SIRT3^EC^KO mice (Figure 5b–d). The myogenic tone in MCA of female SIRT3^EC^KO mice was significantly impaired when the perfusion pressure was between 50 and 70 mmHg (Figure 5e). There were no significant differences in MCA wall tension, distensibility, and stiffness between male and female SIRT3^EC^KO mice (Figure 5f–i). Female control SIRT3^LoxP^ mice had less active diameter changes than males under the pressure of 30–90 mmHg (Figure S9a). There were also no significant differences in MCA mechanical properties between male and female control SIRT3^LoxP^ mice (Figure S9b–d,g–i), except the wall tension and myogenic tone that were slightly different when the perfusion pressure was increased from 110 to 150 mmHg in female control SIRT3^LoxP^ mice compared to male control SIRT3^LoxP^ mice (Figure S9e,f).

## 4 DISCUSSION

In this study, we demonstrated for the first that the deficiency of mitochondrial SIRT3 in EC leads to impairment of myogenic response in CA and MCA, which is essential for modulating vascular tone and ensuring proper blood flow in response to changing perfusion pressure. Furthermore, knockout of endothelial SIRT3 reduced Ach‐induced EDR and SNP‐induced EIR in a sex and organ‐specific manner. These novel findings demonstrate that endothelial mitochondrial SIRT3 uniquely affects the pressure‐flow‐induced changes in diameter—the myogenic response—of isolated mouse intramural coronary and cerebral arteries. Taken together, our results suggest that endothelial SIRT3 is necessary for maintaining vascular tone and pressure‐induced myogenic response.

The myogenic response is an important mechanism involved in the local regulation of blood flow in various organs (Cui et al., 2022, 2023; Petersen et al., 2002). The myogenic response which induces vessel contraction or dilation in response to changed intravascular pressure, has been shown to play an important role in regulating myocardial blood flow (DeFily & Chilian, 1995; Johnson et al., 2021; Linden & Losano, 1991). A previous study suggests that myogenic responses of smaller arterioles have a greater impact than those of larger arterioles on the local regulation of blood flow (Szekeres et al., 2001). Due to technical difficulties, few studies addressed the characteristics of the myogenic response and its modulation in the intramural coronary arterioles. SIRT3 has been implicated in the maintenance of mitochondrial function, which has a critical role in cellular metabolism, aging, and stress resistance (Giralt & Villarroya, 2012; McDonnell et al., 2015). SIRT3‐deficient mice exhibit an accelerated aging progression (McDonnell et al., 2015; Park et al., 2011; Porter et al., 2014). Aging has also been shown to decrease the myogenic response of coronary arteries (Shipley & Muller‐Delp, 2005). We demonstrated the critical roles of endothelial SIRT3 in regulating coronary microvascular dysfunction, diastolic dysfunction, and cardiac recovery post‐myocardial ischemia (He et al., 2016, 2017, 2019). The myogenic response is an intrinsic property of vascular smooth muscle cells in small arteries and arterioles that allow these small vessels to constrict in response to elevated intraluminal pressure and dilate when the internal pressure is reduced. Although a previous study revealed that intramural coronary arterioles display significant myogenic responses which are modulated by nitric oxide (Szekeres et al., 2004), the direct contribution of endothelium to myogenic response has not been studied extensively in intramural coronary arterioles. In this study, we aim to examine the characteristics and physiologic role of myogenic responses in coronary arterioles embedded in the left ventricular wall in endothelial SIRT3 knockout mice. Our data showed that the myogenic responses were significantly impaired in distal intramural branches of the left anterior descending coronary artery of male and female SIRT3^EC^KO mice. These results further validate the critical role of endothelial cells and mitochondrial dysfunction in regulating myogenic response and coronary blood flow. In addition, impairment of coronary myogenic response and disruption of myocardial blood perfusion may also be one mechanism responsible for pressure overload‐induced heart failure in SIRT3^EC^KO mice as we reported previously (Zeng et al., 2020b).

In addition, a very similar phenomenon of impaired myogenic response was observed in the MCA of SIRT3^EC^KO mice further supporting the critical role of endothelial cells in the regulation of vessel myogenic response in response to changing perfusion pressure. MCA exhibits the myogenic response, which can decrease or increase the diameter as MCA lumen pressure is increased or decreased (Palomares & Cipolla, 2014). Myogenic response in MCA contributes to the autoregulation of cerebral blood flow and prevents cerebral vascular damage from variations in blood pressure (Palomares & Cipolla, 2014). Impairment of myogenic reactivity and disruption of cerebral vessel autoregulation leads to cognitive decline and stroke (Palomares & Cipolla, 2014), (Pétrault et al., 2019). Taken together, our study demonstrated a potential mechanism for how mitochondrial SIRT3 deficiency in endothelium may promote heart failure and cognitive impairment in aging.

One interesting finding from this study was the significant impairments of endothelial‐independent relaxation in distal intramural branches of the left anterior descending coronary artery and middle cerebral artery of SIRT3^EC^KO mice. Specifically, the differences between EC‐dependent and EC‐independent relaxation suggest a unique regulatory role of endothelial SIRT3 not only in vascular endothelium but also in the vascular smooth muscle cells (VSMCs). The reductions of EIR in SIRT3^EC^KO mice indicate a potential crosstalk between EC and VSMCs. Our previous study has shown a significantly decreased release of apelin in SIRT3^EC^KO mice compared to control mice (Zeng et al., 2020b). Apelin has been shown to relax coronary arteries via the release of nitric oxide (NO) (Anto et al., 2022). Endothelial SIRT3 may interfere with VSMC by releasing growth factors, such as apelin. This supports our hypothesis that endothelial SIRT3 may modulate VSMC function in the distal intramural branches of the coronary artery through apelin signaling pathways via paracrine action. Importantly, the impairment of the EIR and VSMC function, especially in MCA, implies a disruption of autoregulation in blood flow in the cerebral circulation, which could have severe consequences for cerebral perfusion and may promote the progression of cerebral diseases and stroke.

In this study, we found that Ach‐induced EDR was significantly reduced only in the MCA of male SIRT3^EC^KO mice. Surprisingly, SNP‐induced EIR was impaired in the MCA of both male and female SIRT3^EC^KO mice. In CA, EDR was impaired in both male and female mice, whereas EIR was only reduced in the female SIRT3^EC^KO mice. In the comparison of sex differences, we further found that male SIRT3^EC^KO mice had profound vascular dysfunction in MCA. In contrast, female mice had worsened myogenic response and mechanical properties. In CA, vascular dysfunction is predominant in the female SIRT3^EC^KO mice, whereas male mice had profound alterations to vascular mechanical properties. These differences in vascular endothelial function, myogenic reactivity, and mechanical properties between male and female SIRT3^EC^KO mice and between different vascular beds provide another interesting insight into the possible sex and organ‐specific roles of SIRT3 in regulating vascular function and blood flow autoregulation. These findings are critical and warrant further investigation to understand the potential mechanism of sex difference of SIRT3 in blood vessels and different vascular beds. Our study may pave the way for developing sex‐specific therapeutic strategies and understanding the diverse impacts of endothelial dysfunction on different vascular beds and blood flow autoregulation. Further investigation is necessary to define the mechanistic relationship between endothelial SIRT3 and vascular remodeling. It is also worth exploring whether these observations are a direct effect of SIRT3 deficiency in ECs or a compensatory mechanism of endothelial SIRT3 deficiency during development over the 12–15 months lifespan of the mice.

Although we provided important evidence for understanding the role of endothelial SIRT3 in vascular function and myogenic response, there are still some limitations that need to be further addressed. It would be important to elucidate the molecular mechanisms of endothelial SIRT3 on myogenic response via paracrine or autocrine. Additionally, the influence of different ages could be another key factor for vasculature, which should be investigated. As aging is known to affect endothelial cell metabolism, vascular stiffness, and cardiovascular function. Investigating how age‐related changes modulate the role of endothelial SIRT3 could provide deeper insights into age‐specific therapeutic strategies and improve our understanding of the association between aging and vascular function and its impact on cardiovascular diseases. Moreover, the clinical relevance of these findings and the translational research on human vascular biology should be addressed in future studies. Given that endothelial dysfunction is a hallmark of numerous cardiovascular pathologies, comprehending the crucial roles of molecules like mitochondrial SIRT3 in maintaining vascular hemostasis and blood flow autoregulation could provide novel therapeutic strategies for human cardiovascular diseases.

## 5 CONCLUSION

This study reveals the pivotal role of endothelial mitochondrial SIRT3 in maintaining microvascular function and blood flow autoregulation, particularly focusing on pressure‐induced myogenic response, sex, and organ differences of myogenic reactivity. Our findings open new avenues for exploring SIRT3 as a potential therapeutic target in vascular pathologies and associated end organ damages, particularly in cardiovascular and cerebrovascular diseases.

## AUTHOR CONTRIBUTIONS

J.X. Chen wrote the manuscript; J Zhang performed the research and analyzed the data; H. Zeng designed the research and edited the manuscript; Y. Chen contributed thoughtful comments on the project and edited the manuscript, and all authors have read and approved the final manuscript.

## FUNDING INFORMATION

This work was supported by University of Mississippi Medical Center Intramural Research Support Program (IRSP, H.Z.) and the National Institute of General Medical Sciences and the National Heart, Lung, and Blood Institute (R01HL151536, JX Chen).

## CONFLICT OF INTEREST STATEMENT

The authors declare no conflicts of interest regarding this work.

## ETHICS STATEMENT

All protocols were approved by the Institutional Animal Care and Use Committee at UMMC (Protocol ID: 1564, 1189) and were in compliance with the National Institutes of Health Guide for the Care and Use of Laboratory Animals (NIH Pub. No. 85‐23, Revised 1996).

## Supporting information


Data S1.

## Data Availability

Data available within this article or its supplementary materials. The authors confirm that the data supporting the findings of this study are available within this article and its supplementary materials.
