# Supplementary material for: Knockout of Sirtuin 3 in endothelial cells impairs endothelial‐dependent relaxation and myogenic response in mice
Source: Physiol Rep. 2024 Oct 19;12(20):e70060. doi: 10.14814/phy2.70060 (PMC11489619; doi:10.14814/phy2.70060)
Supplement: Supplementary file 1 — Data S1. [file PHY2-12-e70060-s001.pdf]

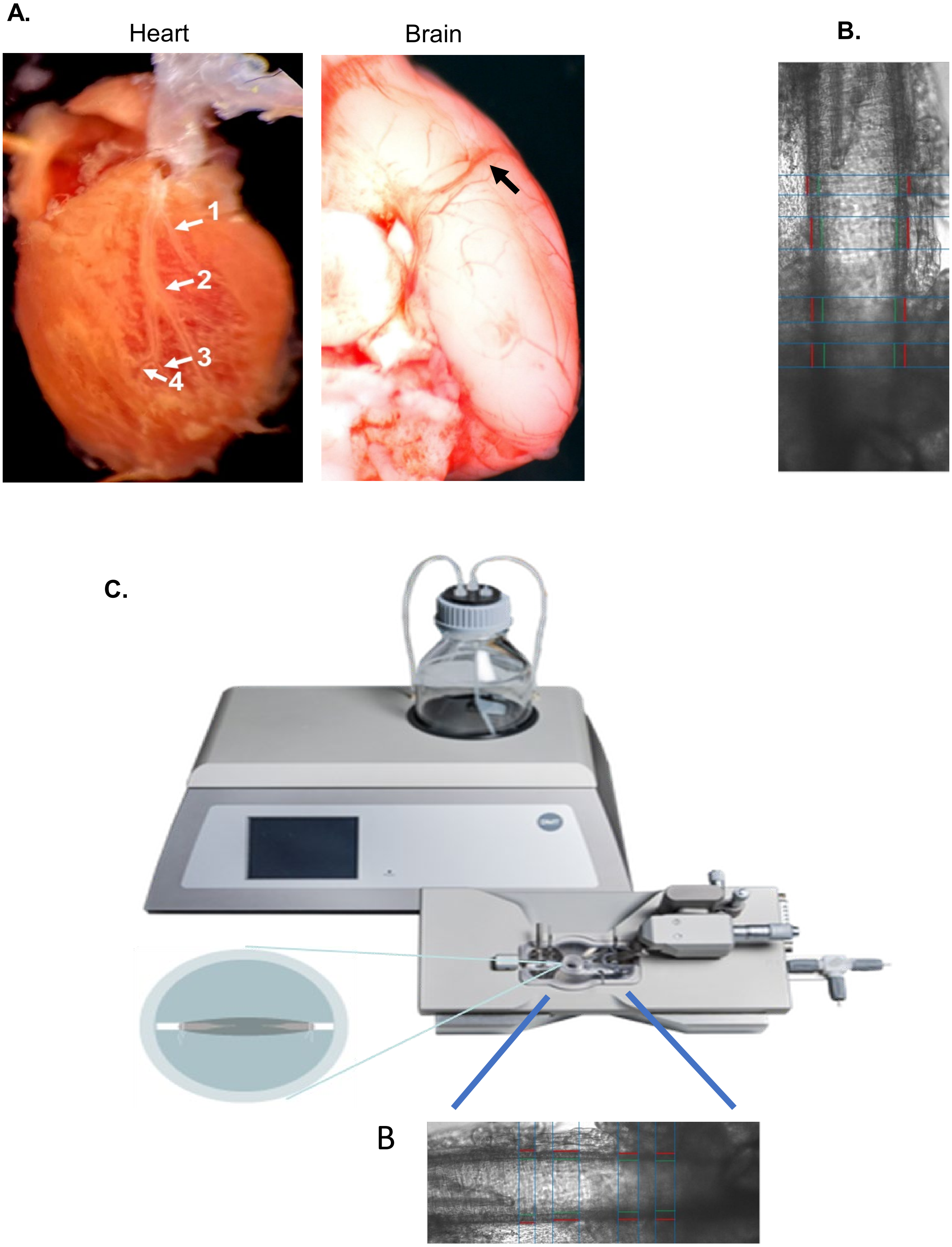

Supplementary Figure 1: A. The distal intramural branches of the heart's left anterior descending coronary artery (CA) or the middle cerebral artery (MCA) were dissected. **B-C,** The fourth branches of CA or mid-terminal of MCA were isolated and mounted on the glass microcannulas in a chamber of myograph system 114P.

Mechanical Properties of Coronary Arterioles (CA)

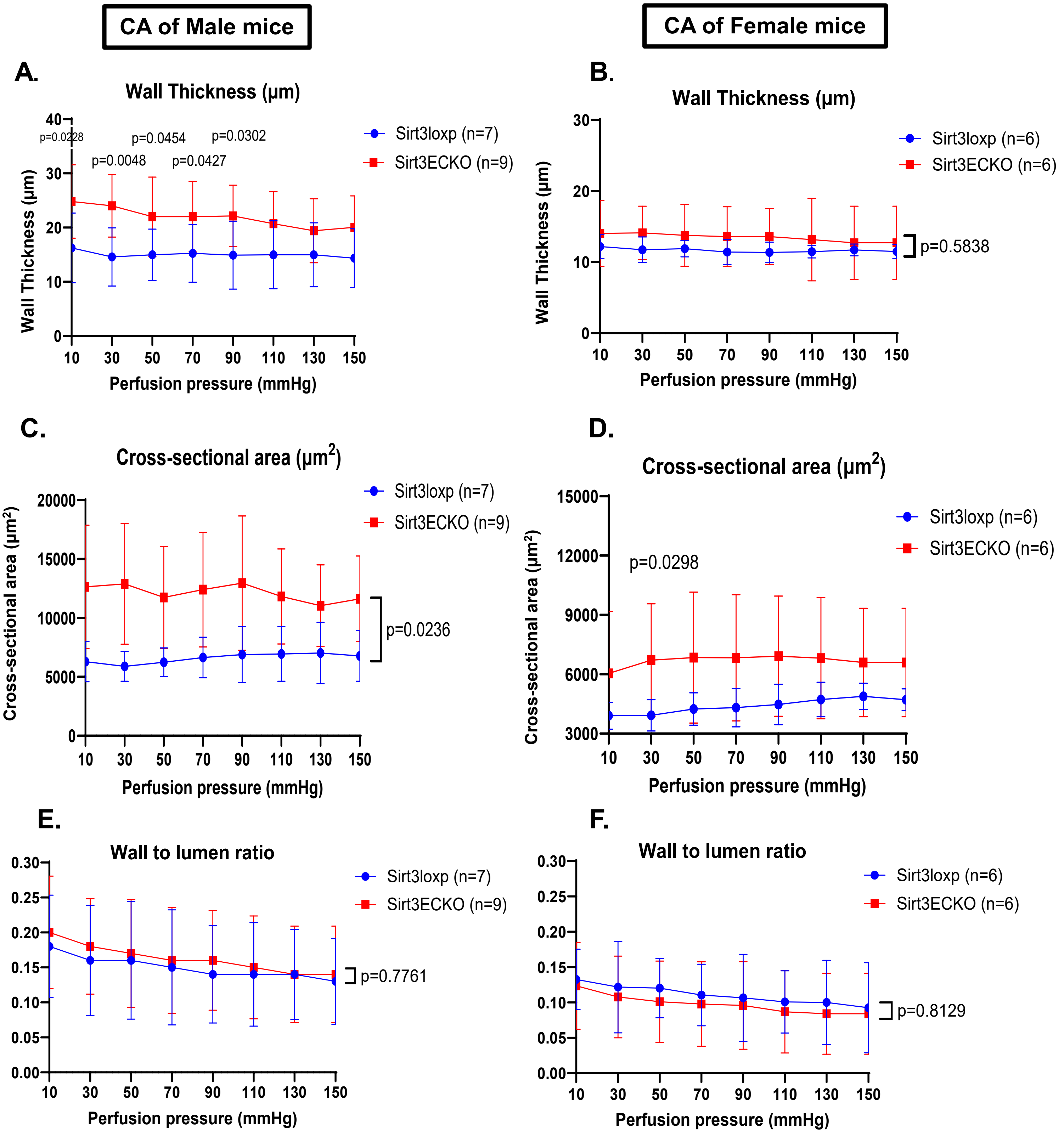

Supplementary Figure 2. A-D: Both the wall thickness and cross-sectional areas of CA were significantly increased in male but not in female Sirt3ECKO mice compared to the respective control Sirt3loxp mice. **E-F:** The arterial wall-to-lumen ratio was not a difference between Sirt3ECKO and control Sirt3loxp in male or female mice (n=6-9 mice, Mean ± SD)

Mechanical Properties of Coronary Arterioles (CA)

CA of Male mice **CA of Female mice**

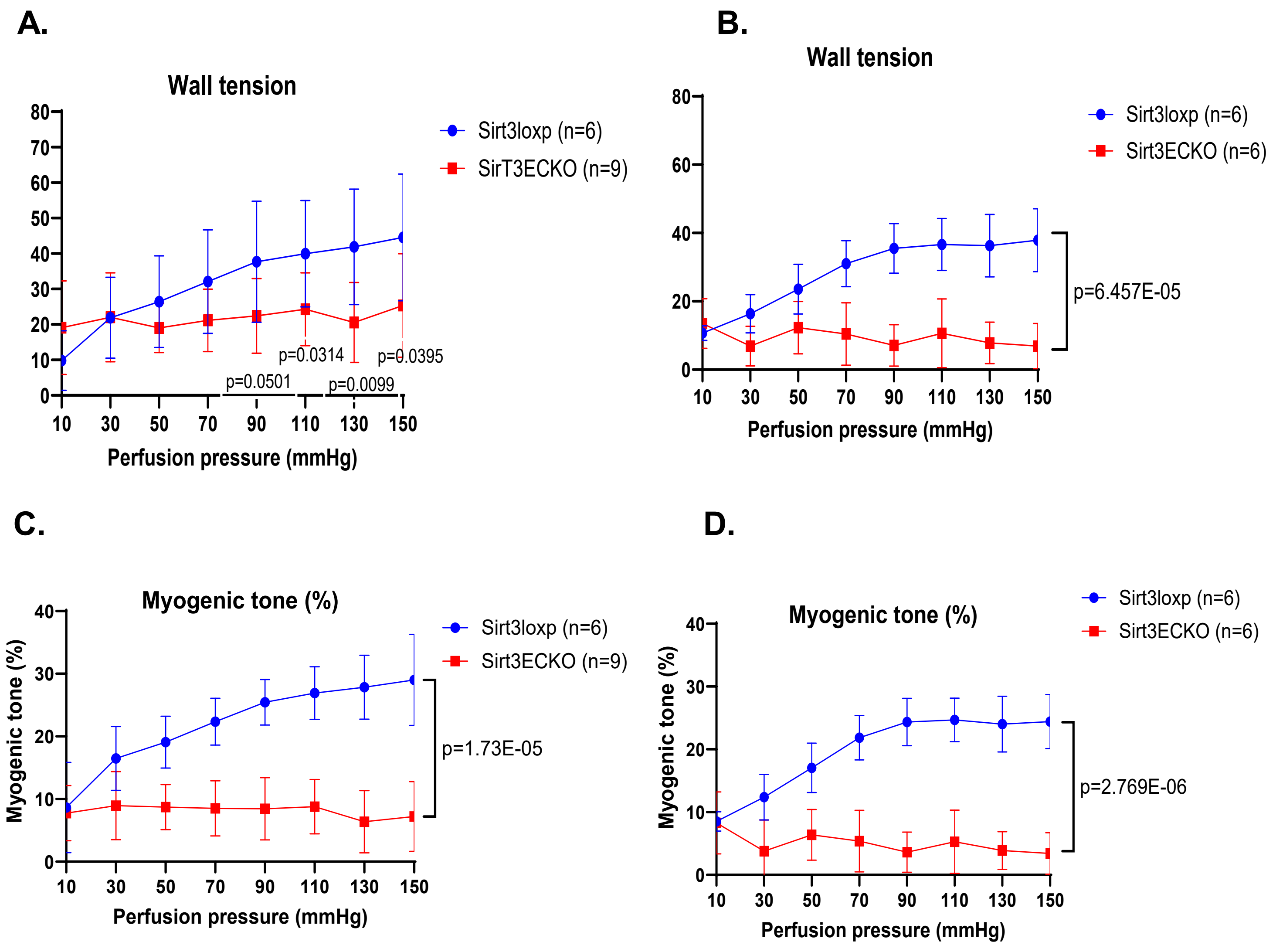

Supplementary Figure 3. A-D. Both the wall tension and the myogenic tones in CAs were significantly decreased in Sirt3 ECKO male or female mice compared to the respective control Sirt3loxp mice. (n=6-9 mice , Mean ± SD)

Mechanical Properties of Coronary Arterioles (CA)

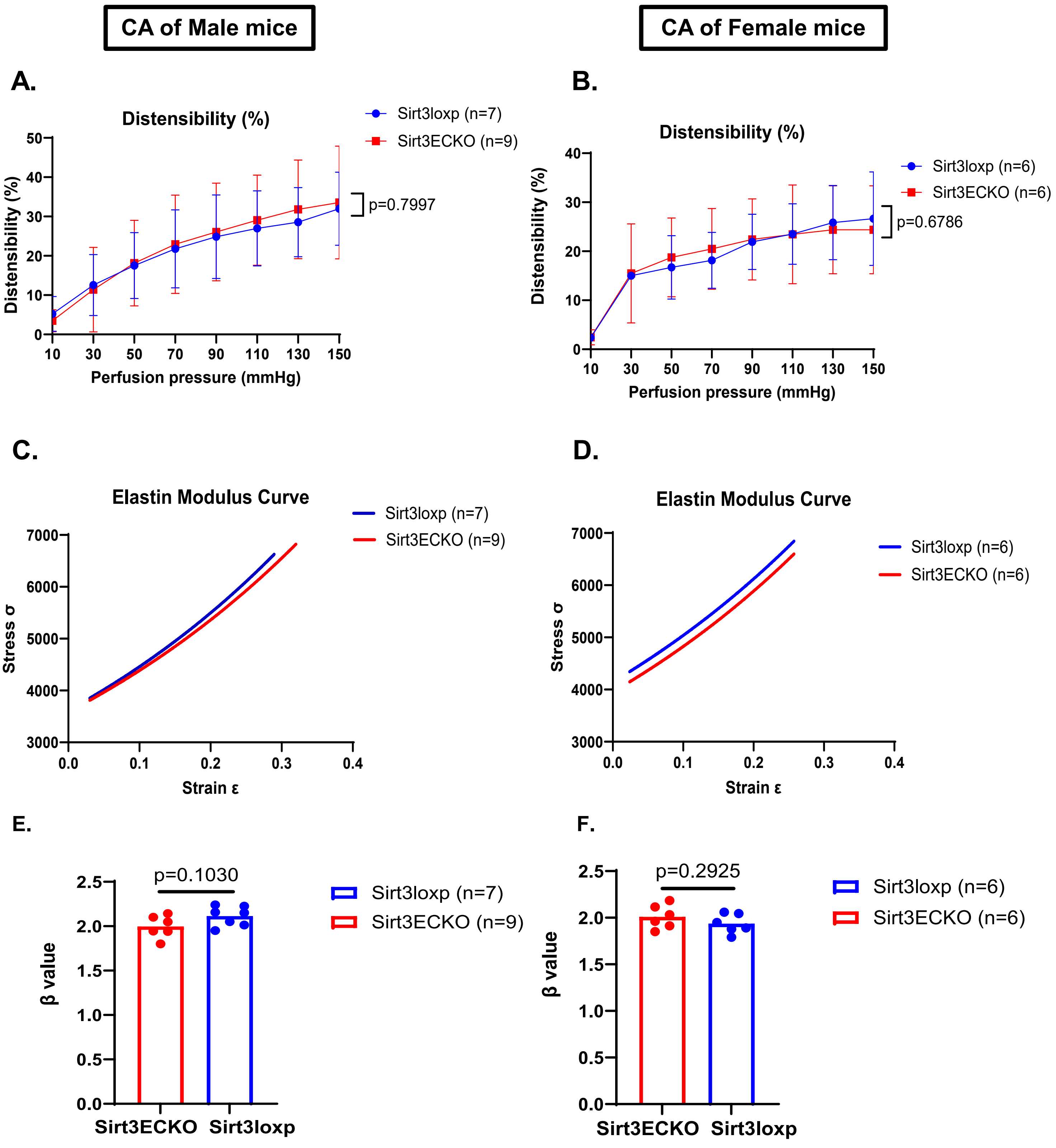

Supplementary Figure 4. A-E. The distensibility, the elastin modulus curve (stress-strain relationship), and the slope of the elastin modulus curve (β value) were not changed between SIRT3 ECKO and control Sirt3loxp in male or female mice. This indicated these properties of Coronary Arterioles (CAs) were no different from normal or defective sirt3 in endothelial cells in mice. (n=6-9 mice, Mean ± SD)

Mechanical Properties of middle cerebral arteries (MCA)

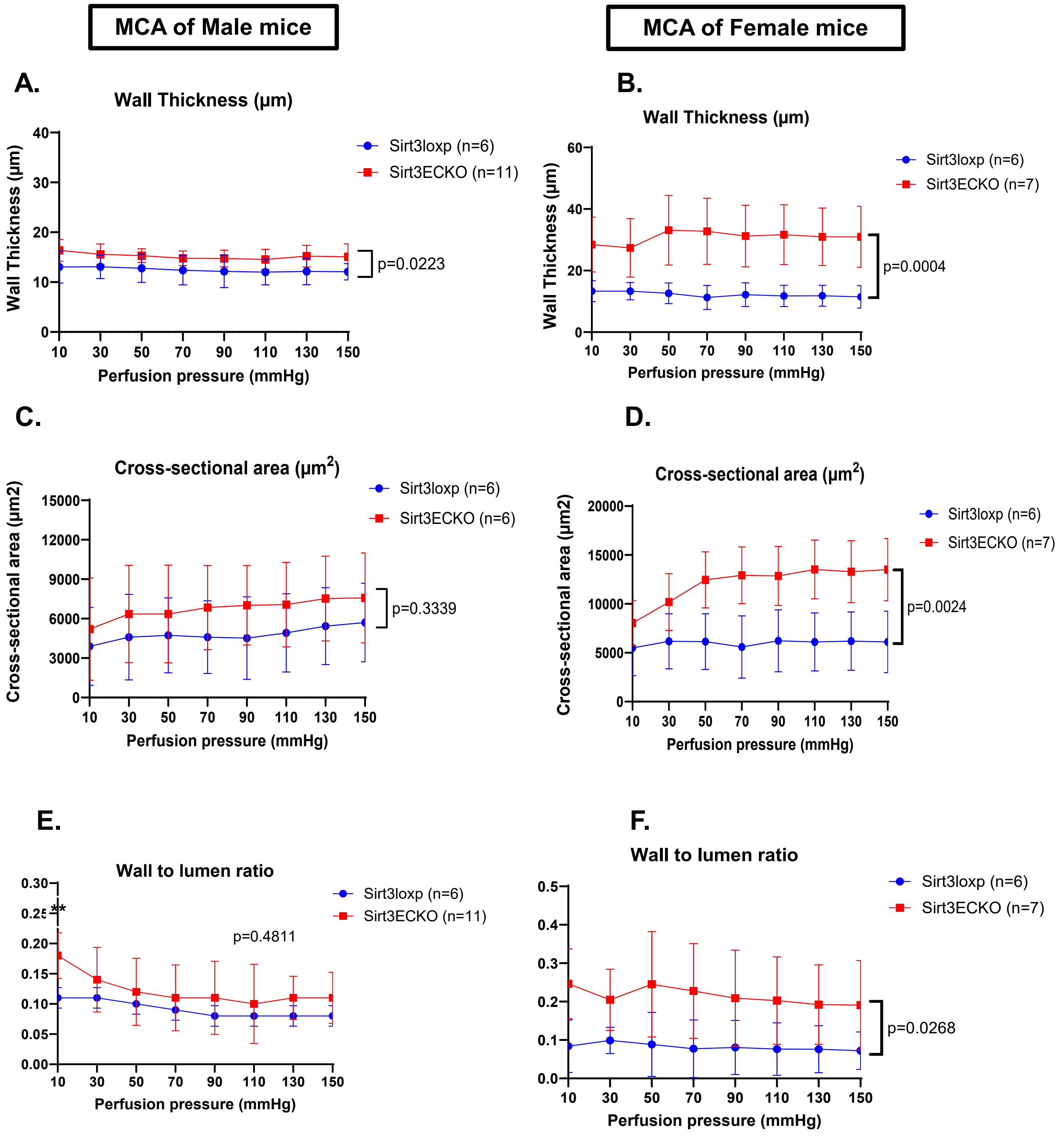

Supplementary Figure 5. A-B. The wall thickness of MCA were significantly increased in male or female Sirt3ECKO mice compared to the respective control Sirt3loxp mice. **C-D.** The cross-sectional areas of MCA were significantly increased in female but not in male Sirt3ECKO mice compared to the respective control Sirt3loxp mice. **E-F:** The arterial wall-to-lumen ratio of MCA was significantly elevated in female Sirt3ECKO but not in male mice than respective Sirt3loxp mice. (n= 6-11 mice, Mean ± SD)

Mechanical Properties of middle cerebral arteries (MCA)

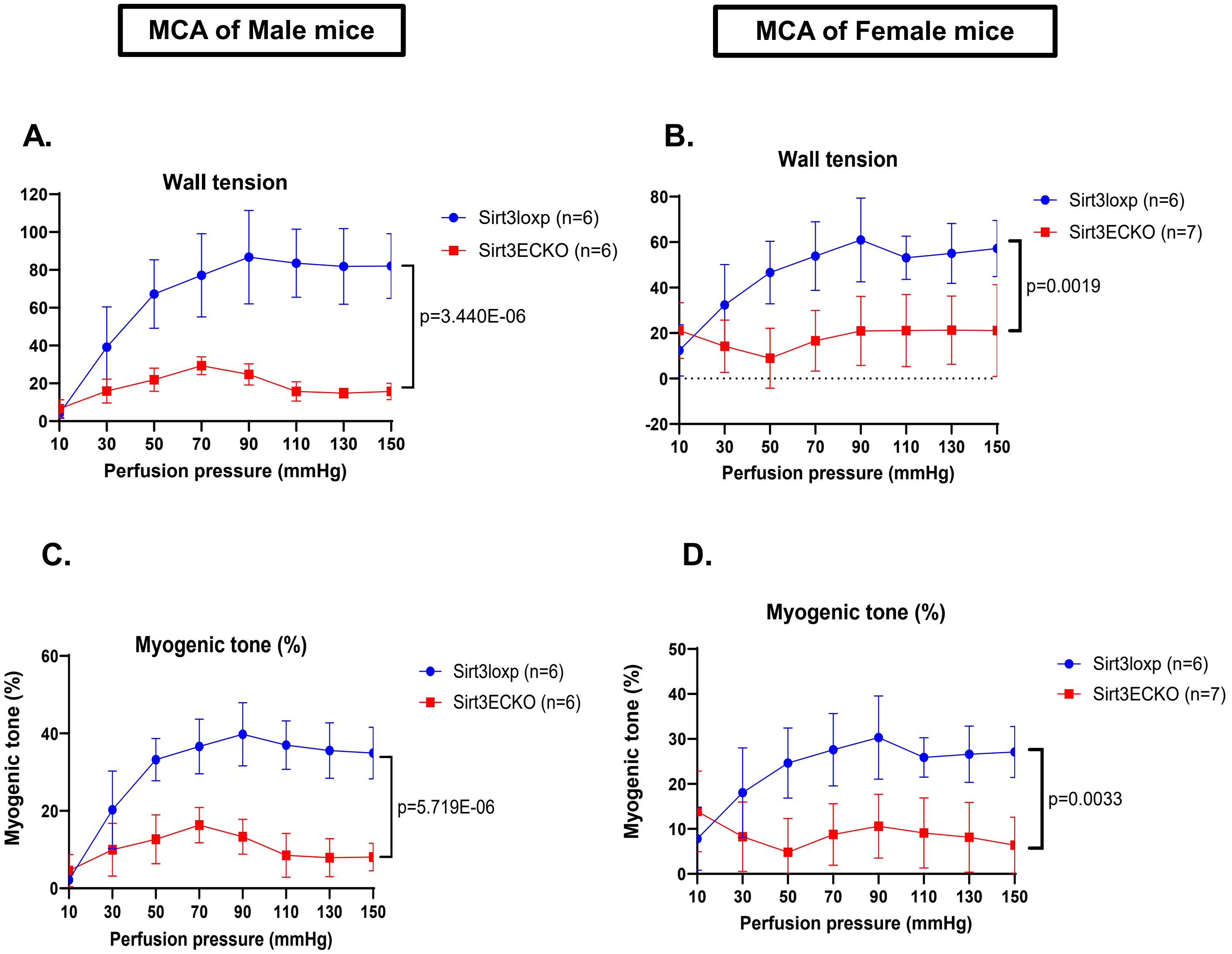

Supplementary Figure 6. A-D. Both the wall tension and the myogenic tones (%) in MCAs were significantly decreased in Sirt3ECKO male and female mice compared to the respective Sirt3loxp mice. (n=6-7 mice, Mean ± SD)

Mechanical Properties of middle cerebral arteries (MCA)

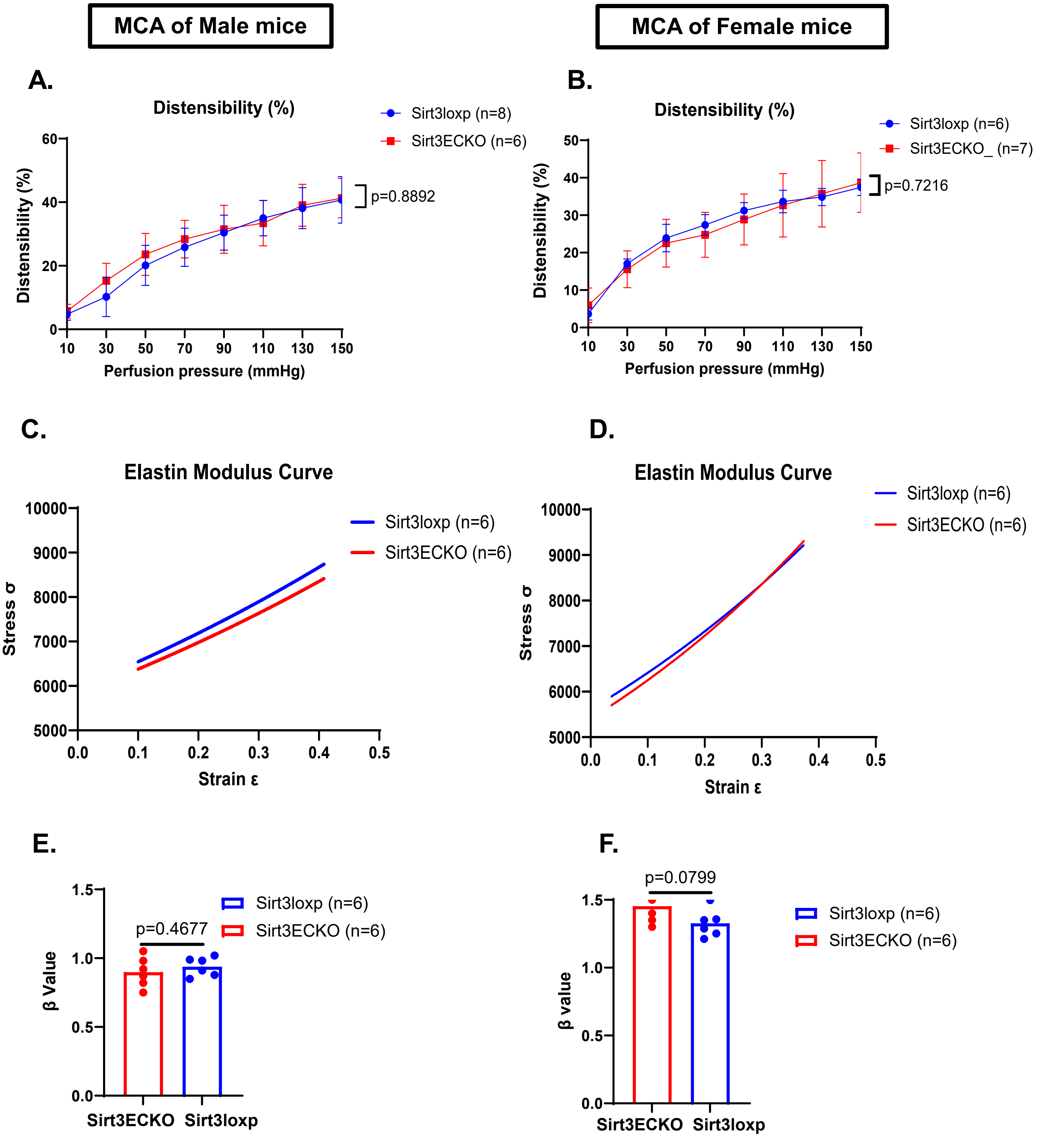

Supplementary Figure 7. A-E: The distensibility, the elastin modulus curve (stress-strain relationship), and the slope of the elastin modulus curve (β value) were not changed between SIRT3 ECKO and control Sirt3loxp in male or female mice. This indicated these properties of middle cerebral arteries (MCAs) were no different from normal or defective sirt3 in endothelial cells in mice. (n=6-7 mice, Mean ± SD)

Mechanical Properties of Coronary Arterioles (CA)

Sirt3loxp mice (Male vs. Female)

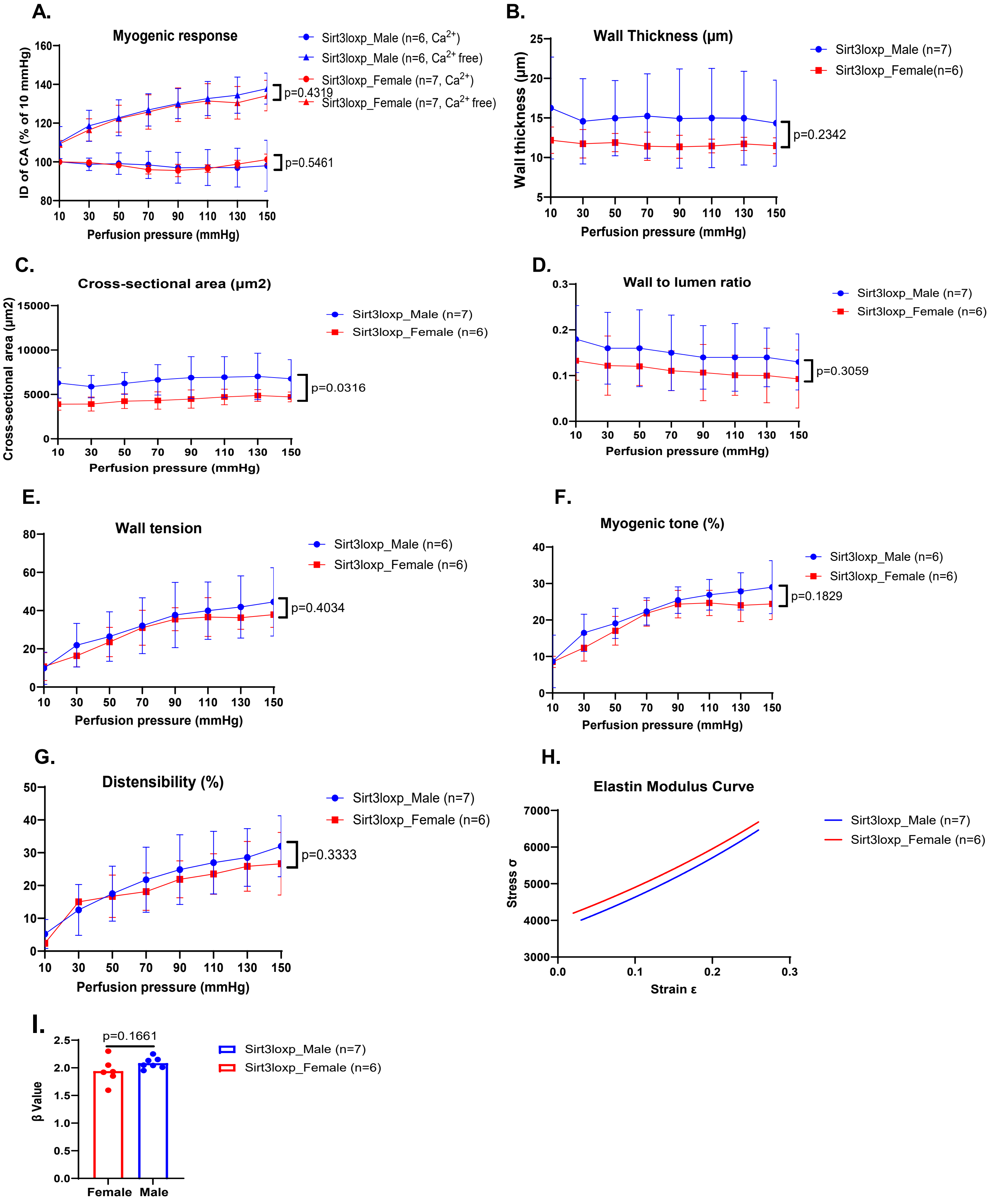

Supplementary Figure 8. A-I: There were no significant differences in myogenic response and vascular mechanical characteristics in CAs between male and female Sirt3loxp mice except that cross-sectional areas of CAs were higher in Sirt3loxp male mice. (n=6-7 mice, Mean ± SD)

Supplementary Figure 8

Mechanical Properties of middle cerebral arteries (MCA)

Sirt3loxp mice(Male vs. Female)

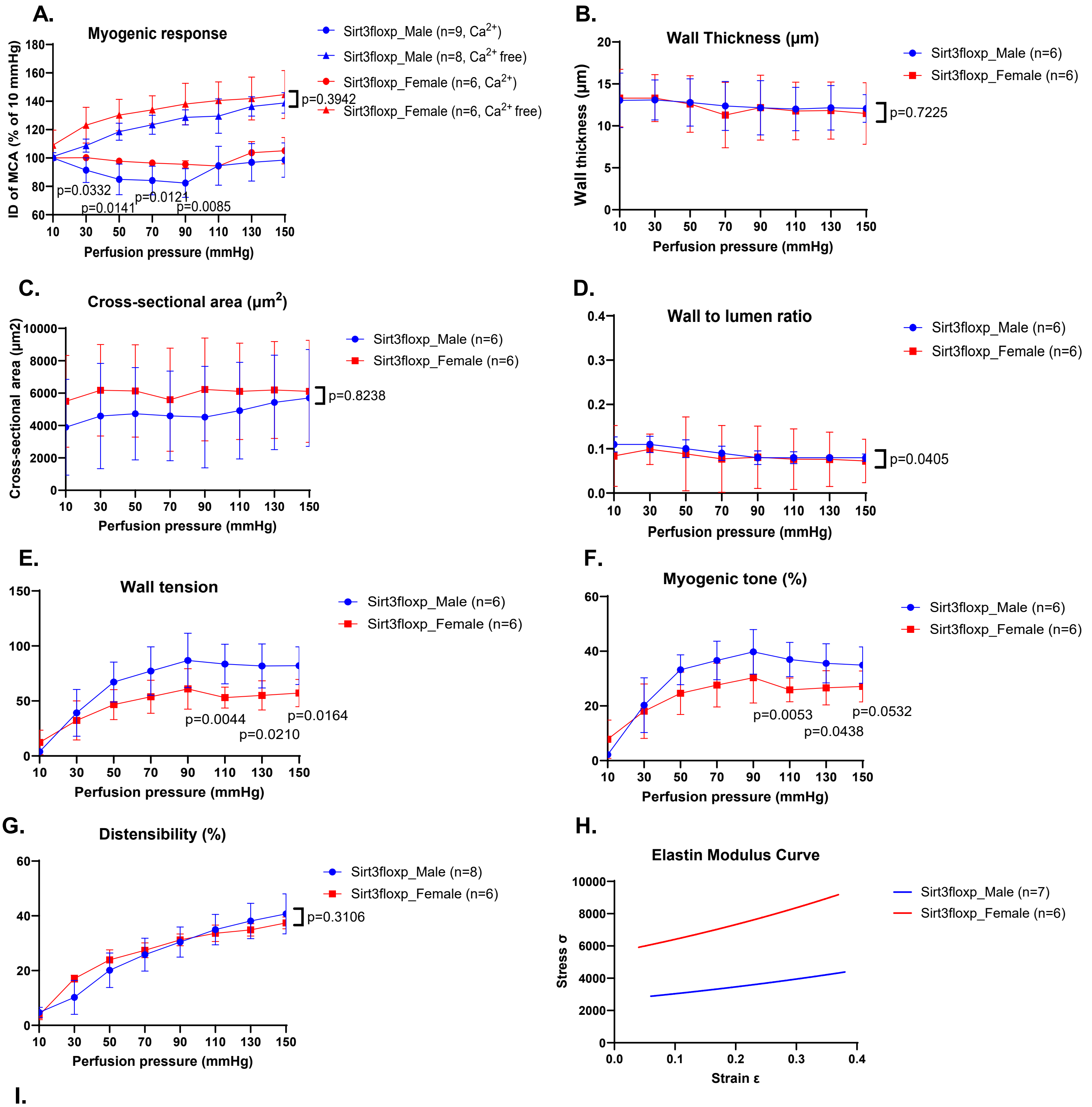

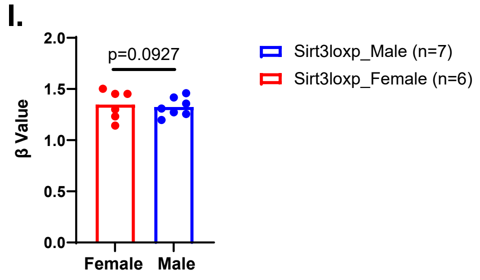

Supplementary Figure 9. A. The myogenic response of MCAs was better in control Sirt3loxp male than female mice between 30 and 90 mmHg of perfusion pressure. The integrity of MCAs was no different between control Sirt3loxp male and female mice in the calcium-free buffer. **B, C, D, G, H, I:** No significant differences in wall thickness, The cross-sectional area, wall-to-lumen ratio, distensibility(%), and stiffness in MCAs of male and female SIRT3loxp mice. **E, F:** The wall tension and myogenic tone(%) were higher in MCA of control Sirt3loxp male mice than female mice between 110 to 150 mmHg of perfusion pressure.. (n=6-8 mice, Mean ± SD)
